# Lack of effect of cyclic adenosine monophosphate modulation during prematuration in vitro on development to the blastocyst stage and expression of specific genes associated with lineage commitment

**DOI:** 10.3168/jdsc.2024-0625

**Published:** 2024-10-11

**Authors:** Raquel Desenzi, Camila J. Cuellar, Quinn A. Hoorn, André Mariano Batista, Rafael Artur da Silva, Peter J. Hansen

**Affiliations:** 1 Department of Animal Sciences and D.H. Barron Reproductive and Perinatal Biology Research Program, University of Florida, Gainesville, FL 32611-0910; 2 Departamento de Medicina Veterinária, Laboratório de Biotécnicas Aplicadas à Reprodução, Universidade Federal Rural de Pernambuco, Recife, PE, Brasil

## Abstract

Pharmacological elevation of cyclic AMP (cAMP) of cultured cumulus-oocyte complexes (COC) before or coincident with initiation of maturation has been reported to improve outcomes for various systems for in vitro production of embryos. Here it was hypothesized that artificial elevation of cAMP in the oocyte for a 2-h period of prematuration would improve developmental competence of matured oocytes and result in increased blastocyst yield and altered expression of genes important for embryonic differentiation. Treated COC were cultured for 2 h with dibutyryl cAMP (dbcAMP), a membrane-permeable form of cAMP, and 3-isobutyl-1-methylxanthine (IBMX), which inhibits phosphodiesterases that convert cAMP to ATP. Subsequently, oocytes were matured and fertilized and the resultant embryos cultured for 7.5 d. There was no effect of treatment on the percent of oocytes or cleaved embryos becoming blastocysts or on transcript abundance of *EOMES*, *GATA4*, *NANOG*, *SOX2*, or *SOX17* in the blastocyst. Results do not support the use of pharmacological enhancers of cAMP concentrations in the oocyte for improving the blastocyst yield following in vitro oocyte maturation, fertilization, and embryo culture.

## Highlights

• Reports show that increasing cAMP in oocytes improved in vitro embryo production.
• That hypothesis was tested by using 2 pharmacological agents to increase cAMP.
• Treatment did not increase the yield or gene expression of blastocysts.

## Graphical Abstract

**Figure:**
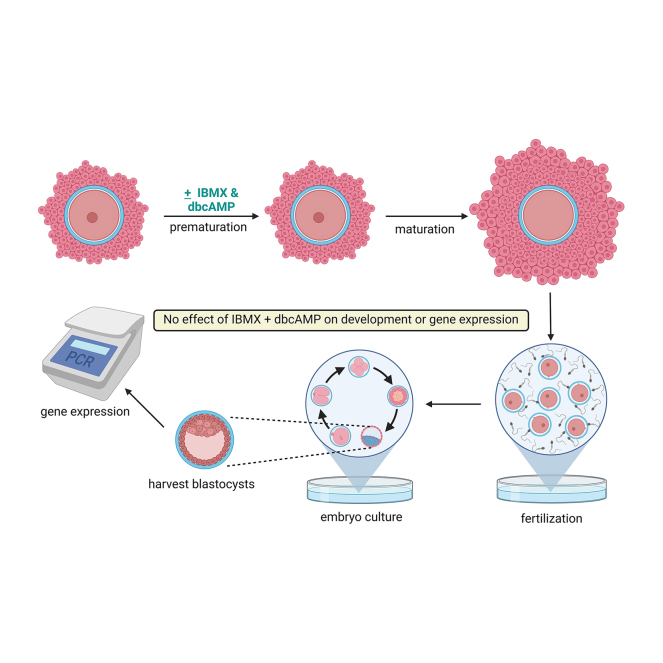
**Summary:** Lack of an effect of 3-isobutyl-1-methylxanthine (IBMX), which inhibits phosphodiesterases that convert cyclic adenosine monophosphate (cAMP) to adenosine triphosphate, and dibutyryl cAMP (dbcAMP), a membranepermeable form of cAMP, added during a 2-hour prematuration period, on subsequent yield or gene expression of blastocysts. Created with BioRender.com.

During the resumption of meiosis, oocytes undergo nuclear and cytoplasmic changes that are crucial for the acquisition of oocyte competence for fertilization and subsequent embryonic development (Fair and Lonergan, 2023). These processes occur differently for oocytes matured in vitro as indicated by reduced competence to form a blastocyst after fertilization (Rizos et al., 2002). Cyclic AMP is one of the important regulators of oocyte maturation; inadequate concentrations of this nucleotide in the oocyte are a proposed cause for reduced developmental competence of oocytes matured in vitro as compared with those matured in vivo (Gilchrist et al., 2016). Pharmacological elevation of cAMP of cultured cumulus-oocyte complexes (**COC**) before or coincident with initiation of maturation improved outcomes for various systems for in vitro production of embryos (Gilchrist et al., 2016; Ramos-Leal et al., 2018; Luciano et al., 2018). In cattle, pharmacological elevation in cAMP has been shown to increase blastocyst yield from oocytes harvested from small follicles (Luciano et al., 1999; Sugimura et al., 2018), small oocytes (Abdel-Ghani et al., 2018), vitrified-warmed oocytes (Ezoe et al., 2015), and cultured follicles (Huang et al., 2013; Chelenga et al., 2023). In contrast to these reports, others have failed to find a benefit of treatments that increase cAMP in the oocyte (Guimarães et al., 2015; Diógenes et al., 2017; Razza et al., 2019).

Here it was hypothesized that artificial elevation of cAMP in the oocyte for a 2-h period of maturation would improve developmental competence of matured oocytes and result in increased blastocyst yield and altered expression of genes important for embryonic differentiation. The approach used to increase oocyte cAMP concentrations was based on that used by Sugimura et al. (2018) and involved treatment with dibutyryl cAMP (**dbcAMP**), a membrane-permeable form of cAMP, and 3-isobutyl-1-methylxanthine (**IBMX**), which inhibits phosphodiesterases that convert cAMP to ATP. In contrast to the hypothesized result, there was no improvement in the proportion of oocytes becoming blastocysts after in vitro maturation, fertilization, and embryo culture. There was also no effect of treatment on blastocyst expression of specific genes involved in lineage commitment.

In the first experiment, embryos were produced by in vitro maturation and fertilization of oocytes harvested from ovaries recovered at a local abattoir using procedures described by Tríbulo et al. (2019). The experiment was performed on 7 occasions (i.e., replicates) using a total of 250 to 300 COC per replicate (n = 1,930 total). The COC were collected from ovaries whose surface was scored with a scalpel blade and the ovary rinsed vigorously in oocyte collection medium (MOFA/BovinePlus with BSA, Minitube USA, Verona, WI). Ovaries selected for COC collection were those that had numerous small (<5 mm in diameter) and medium-sized (5–10 mm) follicles on the surface of the ovary with an absence of larger follicles (∼>10 mm). After washing in collection medium, COC were transferred randomly into groups of ∼10–15 to 50 µL microdrops of prematuration medium (Tissue Culture Medium 199, Gibco Life Technologies, Carlsbad, CA) supplemented with 2 m*M* l-alanyl-l-glutamine (Gibco Life Technologies), 0.2 m*M* sodium pyruvate, 100 units/mL penicillin, 100 µg/mL streptomycin, and 10% (vol/vol) fetal bovine serum (Gibco Life Technologies) covered in cell culture oil (Fujifilm Irvine Scientific, Santa Ana, CA). The COC were randomly assigned to microdrops of control (prematuration medium without additional treatments) or treatment media (prematuration medium containing 0.5 m*M* IBMX [Sigma-Aldrich, St. Louis, MO] and 1 m*M* dbcAMP [Sigma-Aldrich]). Both control and treatment prematuration media contained 0.5% (vol/vol) dimethyl sulfoxide (needed to solubilize IBMX). The COC were incubated for 2 h at 38.5°C in a humidified atmosphere of 5% CO_2_ in air. Thereafter, COC were washed one time in drops of 50 μL oocyte maturation medium (Tissue Culture Medium 199 supplemented with 2 m*M* l-alanyl-l-glutamine, 0.2 m*M* sodium pyruvate, 100 units/mL penicillin, 100 µg/mL streptomycin, 50 ng/mL human recombinant epidermal growth factor [Invitrogen life Technologies, Carlsbad, CA], 5 µg/mL follicle-stimulating hormone [Folltropin, Vetoquinol, Fort Worth, TX], and 10% [vol/vol] fetal bovine serum) and transferred to oil-covered microdrops of 50 μL oocyte maturation medium. The COC were then allowed to complete nuclear maturation for 20 h at 38.5°C in a humidified atmosphere of 5% (vol/vol) CO_2_ in air.

After maturation, fertilization was performed as described by Tríbulo et al. (2019). A pool of frozen-thawed semen from 3 bulls was used for each replicate; the total number of bulls used in the 7 replicates was 4. Sperm were purified using the PureSperm commercial kit (Nidacon, Mölndal, Sweden) with a PureSperm 80% and PureSperm 40% gradient. Purified sperm were suspended to a final concentration of 17 × 10^6^ cells/mL in in vitro fertilization–Tyrode albumin lactate pyruvate (**IVF-TALP**) solution (Tríbulo et al., 2019). Matured COC from each treatment were separately placed in 35-mm Petri dishes containing 1,700 µL of IVF-TALP, 120 µL of sperm, and 80 µL of a penicillamine, hypotaurine, and epinephrine solution (Tríbulo et al., 2019) and incubated for 14 to 18 h at 38.5°C in a humidified atmosphere of 5% (vol/vol) CO_2_ in air.

Putative zygotes (i.e., oocytes exposed to sperm) were denuded to remove cumulus cells by vortexing in hyaluronidase and then placed in groups of 25 to 30 in 50-μL microdrops of synthetic oviduct fluid–bovine embryo 2 (Tríbulo et al., 2019) and incubated in a benchtop incubator (WTA, College Station, TX) at 38.5°C in a humidified atmosphere of 5% (vol/vol) CO_2_. Cleavage was evaluated after 48 h of culture and blastocyst development at d 7.5 after culture. Data on cleavage and blastocyst development were analyzed by logistic regression fit to a binomial distribution using the GLIMMIX procedure of SAS 9.4 (SAS Institute Inc., Cary, NC) with treatment, replicate, and the interaction in the model.

Results on cleavage and development to the blastocyst stage are shown in Table 1. There was no effect of treatment with IBMX and dbcAMP during the period of prematuration on the proportion of putative zygotes that cleaved or on the proportion of putative zygotes or cleaved embryos that became blastocysts at d 7.5 of culture. There was also no effect of treatment on the proportion of blastocysts that were hatching or hatched at d 7.5.

**Table 1:** Effect of prematuration with IBMX and dbcAMP on cleavage and development to the blastocyst stage^1^

Variable	Experiment 1			Experiment 2		
	Control	Treatment	*P*	Control	Treatment	*P*
Cleaved/putative zygotes (%)	84.2 ± 1.3	81.1 ± 1.4	0.3489	81.5 ± 2.9	86.2 ± 2.6	0.4419
Blastocysts/putative zygotes (%)	31.2 ± 1.6	29.5 ± 1.7	0.5914	41.6 ± 3.7	38.3 ± 3.6	0.6383
Blastocysts/cleaved embryos (%)	37.5 ± 1.9	36.7 ± 1.9	0.8152	51.3 ± 4.1	45.0 ± 4.0	0.4728
Hatching and hatched blastocysts/blastocysts (%)	10.5 ± 2.5	8.9 ± 2.2	0.5798	24.6 ± 5.1	13.7 ± 4.3	0.3591

1 Data are LSM ± SEM.

The experiment was repeated a second time using additional conditions except that COC were washed 4 times between prematuration and maturation. The experiment was performed on 3 occasions (i.e., replicates) using a total of 120 COC per replicate (n = 360). Similar results were obtained as for experiment 1; there was no effect of treatment on any variable measured (Table 1).

Quantitative PCR was conducted on 4 pools of 10 blastocysts from experiment 1 for each treatment. Genes analyzed were chosen because they are preferentially expressed in the inner cell mass (*NANOG*, *SOX2*), hypoblast (*GATA4*, *SOX17*), or trophoblast (*GATA4*, *EOMES*; Negrón-Pérez et al., 2017; Isaac et al., 2024). Blastocysts were harvested at d 7.5 of culture, washed with Dulbecco's PBS, treated with acid Tyrode solution to remove the zona pellucida, and stored at −80°C. Extraction of total RNA was performed using the RNeasy Micro Kit (Qiagen, Germantown, MD). After elimination of genomic DNA with DNase treatment, cDNA was produced using the High Capacity cDNA Reverse Transcription Kit (Applied Biosystems, Thermo Fisher, Norwalk, CT). Real-time PCR analysis was carried out on a CFX96 real-time system (Bio-Rad, Richmond, CA) in a reaction containing 1 µL of cDNA, 1 µL of a mix of forward and reverse primers (10 μ*M* each in nuclease-free water), 8 µL of nuclease-free water and 10 µL of SsoFast EvaGreen supermix with low Rox (Bio-Rad, Richmond, CA). Reaction conditions were an initial step of 95°C for 30 s followed by 40 cycles of 5 s at 95°C and 5 s at 60°C. Primers, listed in Table 2, were validated by verifying the standard curve had a correlation coefficient squared (r^2^) > 0.95 and a slope of between −3.0 and −3.5, that there was a single band of expected size upon analysis of amplicons by agarose electrophoresis, and that there was a single peak in the melting curve.

**Table 2:** Primers used in the study

Gene	Primer sequence^1^ (5′–3′)	Annealing temperature (°C)
*SOX2*	F-ACAGTTGCAAACGTGCAAAG	55
	R-AGACCACGGAGATGGTTTTG	
*GATA4*	F-ATCAAAGACACCAGCAGGTCC	57
	R-GACATCGCACTGACCGAGAA	
*GAPDH*	F-GGGTCTTCACTACCATGGAGAA	56
	R-GTTCACGCCCATCACAAACA	
*SOX17*	F-CAGAACCCAGATCTGCACAAC	58
	R-CAGCGTCAGTGCCTTCCA	
*EOMES*	F-GCGGAAAAGCGGACAATAAC	55
	R-TCCAGTGGGAACCAGTGTTA	
*NANOG*	F-GACACCCTCGACACGGACAC	60
	R-CTTGACCGGGACCGTCTCTT	
*YWHAZ*	F-GCATCCCACAGACTATTTCC	53
	R-GCAAAGACAATGACAGACCA	

1 Primer orientation: F = forward; R = reverse.

Transcript abundance was calculated relative to the cycle threshold (**C_T_**) value of the geometric mean of the 2 reference genes: *GAPDH* and *YWHAZ*. Effects of treatment were determined by ANOVA using the GLM procedure of SAS and with treatment and replicate as terms in the mathematical model. Tests of significance are based on delta C_T_ values, and reported LSM ± SEM are based on fold-change relative to the geometric mean of reference genes. Results are shown in Figure 1. There was no effect of treatment on any of the 5 genes examined.

**Figure 1:**
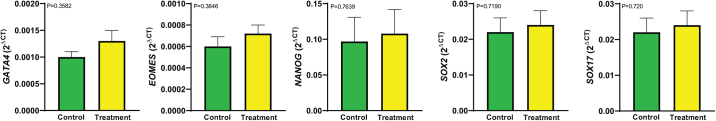
Effect of treatment on transcript abundance for specific genes in the blastocyst. Error bars represent LSM ± SEM.

The results of this study do not support the use of a prematuration regimen designed to increase cAMP concentrations in the oocyte for enhancement of production of embryos in vitro from oocytes harvested from abattoir-sourced ovaries. The experiments in which prematuration with enhancers of oocyte cAMP content have been shown to increase blastocyst yield have used oocytes harvested from small follicles of 2 to 5 mm (Luciano et al., 1999) or 2 to 4 mm in diameter (Sugimura et al., 2018), from small-sized oocytes (Abdel-Ghani et al., 2018), and from follicles that developed in vitro (Huang et al., 2013; Chelenga et al., 2023). Similarly, treatment with either IBMX or forskolin (which activates adenylate cyclase) during maturation of vitrified-warmed oocytes increased blastocyst yield after fertilization (Ezoe et al., 2015). Each of these types of oocytes have poor developmental competence (Otoi et al., 1997; Hendriksen et al., 2000; Ezoe et al., 2015; Chelenga et al., 2023). Perhaps, prematuration in the presence of cAMP enhancers rescues a portion of oocytes with poor developmental competence but has no beneficial effects on more competent oocytes. Consistent with this conclusion are the findings that there was no benefit of treatments that increase cAMP in oocytes harvested from follicles 3 to 8 mm in diameter (Guimarães et al., 2015; Diógenes et al., 2017). In one experiment, the proportion of oocytes from 2 to 6 mm diameter follicles becoming blastocysts was reduced by prematuration with IBMX and forskolin (Razza et al., 2019) and, in another (Guimarães et al., 2015), there was no effect of prematuration with IBMX and forskolin on developmental competence of oocytes from 1 to 3 mm diameter follicles.
